# Identification of the critical replication targets of CDK reveals direct regulation of replication initiation factors by the embryo polarity machinery in *C*. *elegans*

**DOI:** 10.1371/journal.pgen.1008948

**Published:** 2020-12-15

**Authors:** Vincent Gaggioli, Manuela R. Kieninger, Anna Klucnika, Richard Butler, Philip Zegerman

**Affiliations:** 1 Wellcome Trust/Cancer Research UK Gurdon Institute, The Henry Wellcome Building of Cancer and Developmental Biology, University of Cambridge, Cambridge, United Kingdom; 2 Department of Genetics, University of Cambridge, United Kingdom; 3 Department of Biochemistry, University of Cambridge, United Kingdom; NYU School of Medicine, UNITED STATES

## Abstract

During metazoan development, the cell cycle is remodelled to coordinate proliferation with differentiation. Developmental cues cause dramatic changes in the number and timing of replication initiation events, but the mechanisms and physiological importance of such changes are poorly understood. Cyclin-dependent kinases (CDKs) are important for regulating S-phase length in many metazoa, and here we show in the nematode *Caenorhabditis elegans* that an essential function of CDKs during early embryogenesis is to regulate the interactions between three replication initiation factors SLD-3, SLD-2 and MUS-101 (Dpb11/TopBP1). Mutations that bypass the requirement for CDKs to generate interactions between these factors is partly sufficient for viability in the absence of Cyclin E, demonstrating that this is a critical embryonic function of this Cyclin. Both SLD-2 and SLD-3 are asymmetrically localised in the early embryo and the levels of these proteins inversely correlate with S-phase length. We also show that SLD-2 asymmetry is determined by direct interaction with the polarity protein PKC-3. This study explains an essential function of CDKs for replication initiation in a metazoan and provides the first direct molecular mechanism through which polarization of the embryo is coordinated with DNA replication initiation factors.

## Author summary

How and when a cell divides changes as the cell assumes different fates. How these changes in cell division are brought about are poorly understood, but are critical to ensure that cells do not over-proliferate leading to cancer. The nematode *C*. *elegans* is an excellent system to study the role of cell cycle changes during animal development. Here we show that two factors SLD-2 and SLD-3 are critical to control the decision to begin genome duplication. We show that these factors are differently distributed to different cell lineages in the early embryo, which may be a key event in determining the cell cycle rate in these cells. For the first time we show that, PKC-3, a key component of the machinery that determines the front (anterior) from the back (posterior) of the embryo directly controls SLD-2 distribution, which might explain how the polarisation of the embryo causes changes in the proliferation of different cell lineages. As PKC-3 is frequently mutated in human cancers, how this factor controls cell proliferation may be important to understand tumour progression.

## Introduction

Eukaryotes replicate their genomes from multiple origins that fire throughout S-phase of the cell cycle. Programmed changes in the number, timing and position of origin firing occur during differentiation and development across many metazoa [1]. As a result, different cell types exhibit dramatic changes in the rate of S-phase and the timing with which different parts of the genome are replicated. The mechanisms and physiological importance of such changes in genome duplication during the lifetime of an organism are very poorly understood. With its highly stereotypical cell divisions, the early *C*. *elegans* embryo provides an ideal system to study the role of cell cycle control during development. As early as the second embryonic division, polarity cues generate cells with different S-phase lengths [2,3]. Activators of cyclin-dependent kinase (CDK) are asymmetrically distributed in the early embryo [2,4–6] and CDK activity has been shown to be important for determining the synchrony of division [6]. Despite this, how CDKs control embryonic cell cycle length is not known.

CDKs play a critical role in the initiation of DNA replication across eukaryotes [7]. In budding yeast, CDK phosphorylates two essential initiation factors Sld2 and Sld3, which results in their phospho-dependent interaction with the BRCT repeats of Dpb11 [8,9]. This CDK-dependent complex results in the recruitment of additional proteins, such as the leading strand polymerase (Pol ε) and helicase activatory factors, which together allow replisome assembly by a poorly understood mechanism [10]. Phosphorylation of Sld2 and Sld3 and interaction with Dpb11 is sufficient for the function of CDK in replication initiation in yeast, as mutations that drive the interactions between these proteins can bypass the requirement for CDK to initiate replication [8,9].

Importantly, Sld3, Sld2 and Dpb11, together with another replication initiation factor Dbf4 are low abundance and rate limiting for replication initiation in yeast [11,12]. The orthologues of these same factors are also limiting for S-phase length during the early embryonic divisions in *Xenopus* [13]. In *Drosophila*, increasing CDK activity is sufficient to reduce S-phase length in the early embryo [14], although the same is not true in *Xenopus* or zebrafish [15,16]. It would therefore seem that limiting CDK activity and/or low levels of the key CDK substrates, Sld3 and Sld2 and their binding partners might provide a simple mechanistic explanation for how diverse organisms regulate the rate of replication initiation and thus total S-phase length. Unfortunately the testing of this hypothesis has been hampered by the difficulties in identifying the true targets of CDKs in replication initiation in developmental model systems [17].

We have previously provided the first example of an essential CDK substrate required for replication initiation in a metazoan through the characterisation of *C*. *elegans sld-2* [18]. Mutation of the CDK sites in *sld-2* to alanine prevented the interaction with the Dpb11 orthologue MUS-101 (also known as Cut5/TopBP1) and resulted in lethality, while phospho-mimicking mutations in these CDK sites restored the interaction with MUS-101 and restored viability [18]. Having characterised *sld-2* as an essential CDK target in *C*. *elegans* we set out to identify and characterise the Sld3 orthologue in this organism to determine the importance of regulation of both of these substrates during development. In this study we show that phospho-mimicking mutations at the critical CDK consensus sites in *sld-2* and *sld-3* are sufficient to fulfil at least in part the essential functions of cyclin E in *C*. *elegans*. Both SLD-2 and SLD-3 are asymmetrically localised in the early embryo and the asymmetry of SLD-2 is directly regulated by an interaction with the polarity factor PKC-3. This study provides the first direct link between the cell polarity machinery and DNA replication control and pinpoints *sld-2* and *sld-3* as potentially key factors for determining S-phase length in the early embryo in *C*. *elegans*.

## Results

### ZK484.4 is *C*. *elegans* SLD-3

**Fig 1:**
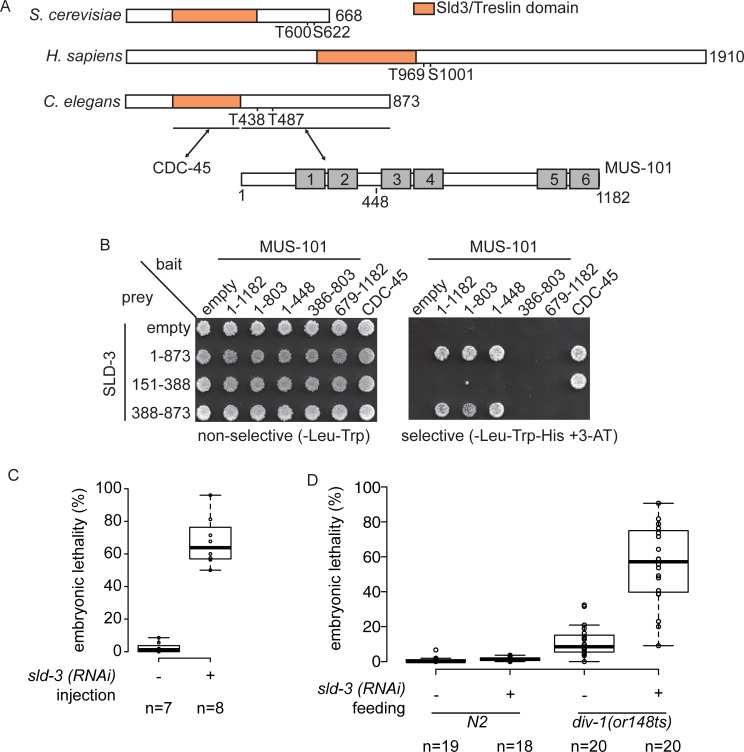
ZK484.4 is *C*. *elegans sld-3* A. Scale diagram of Sld3, Treslin and ZK484.4 from budding yeast, humans and *C*. *elegans* respectively. The Cdc45 interaction (Sld3/Treslin) domain is in orange. The essential CDK sites in Sld3/Treslin and their potential orthologues in *C*. *elegans* are numbered. The regions of interaction between *C*. *elegans* ZK484.4 and CDC-45/MUS-101 are indicated below, together with a scale diagram of MUS-101 showing the 6 BRCT repeats as grey boxes. **B.** Yeast two-hybrid analysis between MUS-101 and CDC-45 bait constructs and SLD-3 prey on non-selective (-Leu-Trp) and selective medium (-Leu-Trp-His+3-AT). **C.** Box and whisker plot of embryonic lethality with and without *sld-3* RNAi by injection. n refers to the number of mothers analysed. **D.** As C except RNAi was performed by feeding at 21°C.

To identify the *C*. *elegans* orthologue of the CDK target Sld3/Treslin, we performed homology searches using the conserved Cdc45 interaction domain (also known as the Sld3/Treslin domain, orange, Fig 1A) [19,20]. From this, we identified ZK484.4 as the best-hit for a potential orthologue of Sld3/Treslin in *C*. *elegans* (Fig 1A). To determine whether ZK484.4 is a functional orthologue of Sld3, we first analysed the interactions of this protein with the replication initiation factors CDC-45 and MUS-101, the *C*. *elegans* orthologue of Dpb11/TopBP1 (S1F Fig is a table of orthologue names). Yeast two-hybrid analysis revealed that the Sld3/Treslin domain of ZK484.4 (151–388) interacted with *C*. *elegans* CDC-45, while the C-terminus of SLD-3 (388–873) interacted with MUS-101 (Fig 1B). Interestingly ZK484.4 interacted with the region of MUS-101 (1–448) encompassing the N-terminal BRCT repeats (Fig 1B), which is the same region of interaction between Sld3/Treslin and the MUS-101 orthologues (Dpb11/TopBP1) in yeast, *Xenopus* and humans [8,9,21,22]. These conserved interactions strongly suggested that ZK484.4 is indeed the *C*. *elegans* orthologue of Sld3/Treslin and we hereby refer to ZK484.4 as *sld-3*. Notably we did not identify an orthologue of the Sld3/Treslin interacting protein Sld7/MTBP [23]. To avoid confusion we hereafter refer to all Sld3 orthologues using the name Sld3 and all Dpb11/TopBP1 orthologues by the name Mus101.

As Sld3 is essential for replication initiation across eukaryotes we set out to test whether *sld-3* is also essential in *C*. *elegans*. RNAi of *sld-3* by injection indeed showed that this is an essential gene (Fig 1C). Consistently with a role for *sld-3* in DNA replication, partial knock down of *sld-3* through RNAi by feeding resulted in synthetic lethality with the *div-1* mutant in the B subunit of polymerase alpha [24] at the semi-permissive temperature (Fig 1D). Together these data confirm that ZK484.4 is likely to be the functional orthologue of *sld-3*.

### *C*. *elegans* SLD-3 has two essential CDK sites

**Fig 2:**
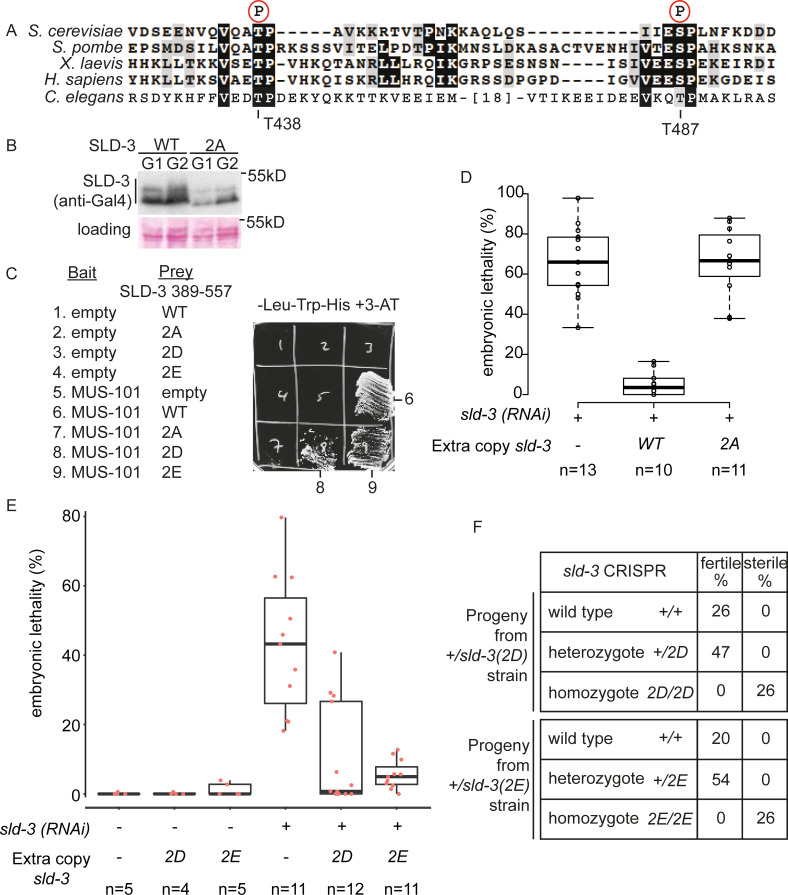
Two CDK sites in SLD-3 are essential for the interaction with MUS-101 A. Alignment of the CDK sites in Sld3/Treslin required for the interactions with Dpb11/TopBP1. The amino acid numbers of the two orthologous CDK sites in *C*. *elegans* SLD-3 are indicated below. **B.** Western blot of Gal4-SLD-3 (389–557) wild type (WT) or the 2A mutant expressed in yeast arrested either in G1 phase (low CDK activity) with alpha factor, or arrested in G2 phase (high CDK activity) with nocodazole. **C.** Yeast two-hybrid analysis between MUS-101 (1–448) and SLD-3 (389–557) wild type (WT) or with the two CDK sites threonine 438 and 487 mutated to alanine (2A), aspartic acid (2D) or glutamic acid (2E). **D** and **E.** Box and whisker plot of embryonic lethality after *sld-3* RNAi by injection as in Fig 1C. The extra, RNAi insensitive copies of *sld-3* are inserted at a MosSCI site and expressed from the *mex-5* promoter. n refers to the number of mothers analysed. **F.** Percentage of progeny from heterozygous *+/sld-3(2D)* (top) or *+/sld-3(2E)* (bottom) parents that were either fertile or sterile. These 2D/2E mutations were generated by CRISPR at the endogenous *sld-3* locus. For 2D n = 38, for 2E n = 91.

Sld3 orthologues are critical CDK substrates in yeast, *Xenopus* extracts and human cells and CDK phosphorylation of Sld3 at two sites mediates the interaction between Sld3 and the N-terminal BRCT repeats of MUS-101 orthologues in these organisms [8,9,21,25]. As *C*. *elegans* SLD-3 also interacts with the N-terminal region of MUS-101 (Fig 1B), we set out to determine whether CDK sites were crucial for this interaction. *C*. *elegans sld-3* has two CDK sites at positions 438 and 487, which show homology to the two essential sites in other Sld3 proteins (Fig 2A). Importantly, a phosphoproteomic study has shown that SLD-3 T438 is phosphorylated *in vivo* in *C*. *elegans* [26] and expression of *C*. *elegans* SLD-3 in yeast resulted in lower mobility forms that were more abundant in cells arrested in G2 phase (with high CDK activity) rather than in G1 phase (with low CDK activity) (Fig 2B), consistent with CDK phosphorylation of SLD-3. Importantly, mutation of these conserved threonines 438 and 487 to alanine (hereafter called the 2A mutant) abrogated the cell cycle-dependent shift in SLD-3 (Fig 2B) and prevented the interaction between SLD-3 and MUS-101 (Fig 2C). To test whether this interaction is important *in vivo*, we inserted in the genome at a MosSCI site an RNAi insensitive copy of either wild type or *sld-3(2A)* fused to mCherry [27]. The expression of these MosSCI alleles was similar, as determined by mCherry fluorescence levels (see for example S3B Fig). Importantly, while the *sld-3* wild type allele fully rescued the *sld-3* RNAi lethality, the 2A mutant that cannot interact with MUS-101 could not rescue this lethality (Fig 2D). This suggests that the two conserved CDK sites (Fig 2A) that are required for interaction with MUS-101 (Fig 2C) are critical *in vivo* in *C*. *elegans*.

To further test the importance of phosphorylation of these two sites in SLD-3 for the interaction with MUS-101 we mutated the two essential CDK sites to aspartic acid (2D) or glutamic acid (2E), which potentially mimics phosphorylation of these sites. Significantly these phospho-mimicking mutants restored the interactions with MUS-101 (Fig 2C). In addition, while *sld-3* RNAi resulted in high levels of lethality as previously shown, an RNAi insensitive copy of *sld-3(2D)* partially rescued this lethality, while the *sld-3(2E)* allele almost fully rescued the loss of wild type *sld-3* (Fig 2E), unlike the situation for human Sld3 [28]. Together this shows that mutations that mimic phosphorylation of *sld-3* at these two essential sites allow MUS-101 interaction and restore viability *in vivo*.

Since expression of the *sld-3(2D)* or *(2E)* mutants as a second copy restored viability after *sld-3* RNAi, we set out to generate these alleles at the endogenous locus by CRISPR. While heterozygotes of the CRISPR-generated *sld-3(2D)* and *(2E)* mutants were viable, the homozygotes were sterile (Fig 2F). We wondered whether instead of mutating both CDK sites, mutation of just one site might be sufficient to generate viable alleles. Strains that were homozygous for either T438 or T487 mutated to alanine resulted in intermediate levels of embryonic death and infertility (S1A–S1E Fig). While mutation of these individual sites to aspartic acid did not rescue the lethality/sterility, mutation of these sites to glutamic acid significantly reduced the lethality exhibited by the alanine mutants (S1A–S1E Fig). Together with the analysis of the *sld-3* alleles as a second copy (Fig 2D and 2E), these data show that while alanine mutants of either of the two, or both CDK sites show lethality, phospho-mimicking mutants can bypass and rescue to some extent this lethality *in vivo*.

We are not sure why the *sld-3(2E)* allele shows high levels of viability after *sld-3* RNAi (Fig 2E), but not as a homozygous allele at the endogenous locus (Fig 2F). One possibility is that constitutive phospho-mimicking of these sites generates phenotypic issues by itself, indeed the CDK bypass mutants of Sld3 alone are sick in yeast [8]. We consider it more likely however that these alleles are simply not fully penetrant in mimicking the essential functions of *sld-3* and therefore behave as hypomorphs, which are viable in the presence of some background level of wild type protein (e.g after RNAi). Despite this, the phospho-mimicking mutants of the CDK sites in *sld-3*, which allow MUS-101 interaction (Fig 2B), show dramatic rescue of *sld-3* RNAi lethality *in vivo* (Fig 2E).

### Bypass of CDK site phosphorylation in SLD-3 and SLD-2 is partially sufficient for cyclin E function

**Fig 3:**
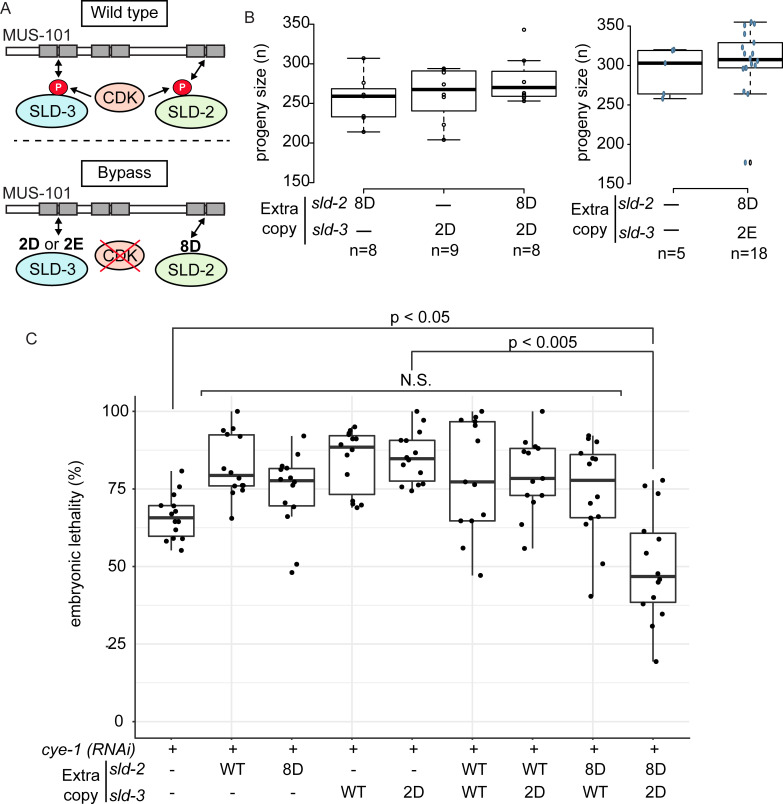
Bypass of CDK site phosphorylation in SLD-3 and SLD-2 is partially sufficient for cyclin E function A. CDK drives the interactions between SLD-2, SLD-3 and MUS-101 (top). The requirement for CDK can be bypassed using phospho-mimicking mutants in the essential CDK sites of SLD-2 (8D) or SLD-3 (2D or 2E). **B.** Box plot of progeny size for the indicated strains containing *sld-2* and/or *sld-3* mutants as extra copies as MosSCI insertions. n refers to the number of mothers analysed. **C**. Box plot of embryo lethality as in Fig 1C after *cye-1* RNAi by feeding in the indicated strains. N.S = non-significant. n = 14 mothers for each condition. P values were calculated using a Mann whitney-Wilcoxon test.

In yeast, phospho-mimicking mutants of Sld2 and Sld3 fulfil the essential functions of CDK in DNA replication initiation [8,9]. In a previous study we characterised the Sld2 orthologue in *C*. *elegans* and identified the *sld-2(8D)* mutant as capable of bypassing the requirement for the CDK sites in *sld-2* to allow interaction with the C-terminus of MUS-101 [18]. Here we have identified the *sld-3(2D)* and *(2E)* mutants that can bypass the requirement for the CDK sites to generate the crucial interaction between SLD-3 and the N-terminus of MUS-101 (Fig 2). Therefore we wondered to what extent the combination of these bypass mutants of *sld-3* and *sld-2* might be able to fulfil essential functions of CDKs in *C*. *elegans* (Fig 3A). As in yeast, we might expect such CDK bypass mutants to be dominant, which is indeed the case for *sld-2(8D)* [18]. Combination of the CDK bypass mutant *sld-2(8D)* with *sld-3(2D)* or *(2E)*, expressed as extra copies at MosSCI sites, resulted in wild type levels of fertility and viability (Fig 3B and S2A–S1B Figs).

Cyclin E/CDK2 is required for the G1-S transition and is responsible for DNA replication initiation, particularly in early embryonic divisions such as in *Drosophila* and *Xenopus* [29,30]. RNAi of Cyclin E (*cye-1*) resulted in embryonic lethality, as expected [31] (Fig 3C and S2C Fig). Expression of the *sld-2* or *sld-3* bypass alleles alone did not restore viability after *cye-1* RNAi (Fig 3C). Importantly combination of both *sld-2(8D)* and *sld-3(2D)* or *(2E)* resulted in significant rescue of viability of *cye-1* RNAi (Fig 3C and S2C Fig). Combination of any of the *sld-2* or *sld-3* MosSCI alleles, even the wild type alleles, with *cye-1* RNAi led to an increase in lethality compared to *cye-1* RNAi alone (Fig 3C). We cannot explain this phenomenon as each MosSCI allele was made independently and outcrossed multiple times. In any case, the combination of the bypass mutants of *sld-2 (8D)* and *sld-3 (2D)* led to significantly reduced lethality, whether we compare to *cye-1* RNAi alone, or *cye-1* RNAi in the other MosSCI allele backgrounds (Fig 3C). This phenotypic rescue by the *sld-2/sld-3* bypass mutants was specific to cyclin E RNAi, as we did not observe any rescue with Cyclin B1 (*cyb-1*) or Cyclin B3 (*cyb-3*) RNAi (S2D and S2E Fig). These data show that *sld-2* and *sld-3* mutants that can bypass the requirement for the critical CDK sites for generating interactions with MUS-101 can fulfil some of the essential functions of Cyclin E *in vivo* in *C*. *elegans*.

### SLD-2 and SLD-3 are asymmetrically localised in the early embryo

**Fig 4:**
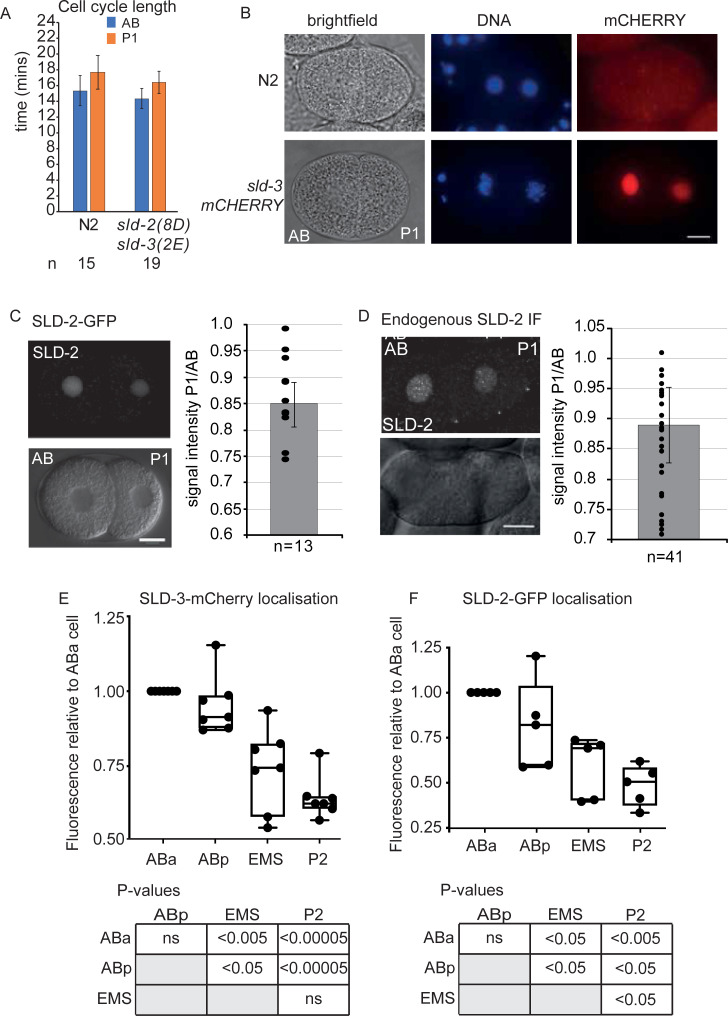
SLD-3 and SLD-2 are asymmetrically localised in the early embryo A. Cell cycle length in the AB and P1 cell of the two-cell embryo in the indicated strains. Error bars are SD. **B.** Images of two-cell embryos of wild type (N2) or containing *mex-5p*::*sld-3-mCherry*::*tbb-2 3-UTR* construct integrated at a MosSCI site. mCherry was visualised by IF, DNA was stained with Hoechst. Scale bar is 10μm. A grey-scale version of this image is available in S3 Fig. **C.** As B. Images of two-cell embryo containing *mex-5p*::*sld-2-gfp*::*tbb-2 3UTR* integrated at a MosSCI site. GFP signal was detected with Confocal Laser Scanning Microscopy. Lower Image shows DIC channel. Scale bar is 10 μm. (Right) Graph indicates the ratio of GFP signal intensity from the P1 cell over the signal from the AB cell. Error bars are 95% CI. **D.** As C, except endogenous SLD-2 was detected by immunofluorescence. **E** and **F**. Quantitation of SLD-3-mCherry signal by IF (E) and SLD-2-GFP signal by fluorescence imaging (F) in the four-cell embryo. The signal in the ABa cell was set to 1. n = 7 for each measurement for E and n = 5 for F. Below the p-values were obtained by paired t-tests, ns is not significant.

During the second embryonic division in *C*. *elegans*, the anterior AB cell has a faster cell cycle than the posterior P1 cell, which is in part due to a shorter S-phase in the AB cell [2]. CDK activity is potentially differentially activated in these two cells due to the asymmetric distribution of CDK regulators, such as *cdc-25* and the cyclin *cyb-3* [4,6]. We wondered to what extent SLD-2 and SLD-3 regulation by CDKs might contribute to this asynchrony of cell division, so we analysed the AB/P1 cell cycle duration using the *sld-2/sld-3* CDK bypass alleles. Fig 4A shows that the duration of the AB and P1 divisions remained very similar in the *sld-2(8D)/sld-3(2E)* mutant relative to wild type, suggesting that CDK phosphorylation of these targets alone is not limiting for S-phase duration in either of these cell divisions.

During this analysis of AB/P1 cycle length using the MosSCI *sld-3* and *sld-2* alleles, which are tagged with mCherry and GFP respectively, we observed that both SLD-3 and SLD-2 showed asymmetric localisation, with more protein in the AB cell nucleus, than P1 (Fig 4B and 4C). This asymmetry was not limited to the MosSCI alleles, as we obtained a similar result using immuno-fluorescence of endogenous SLD-2 (Fig 4D). Interestingly the presence or absence of the essential CDK sites did not affect the asymmetric localisation of SLD-3 (S3A and S2B Fig). The asymmetry we observed was not an artefact of embryo staging, as the difference in abundance of SLD-2 was detected throughout interphase in the two-cell embryo (S3C Fig).

Asymmetric and asynchronous divisions continue beyond the two-cell stage, with the descendants of the AB cell (ABa and ABp) having shorter cell cycles than the descendants of the P1 cell (EMS and P2) with P2 having the longest S-phase of these cells [3,32]. We analysed the abundance of SLD-2 and SLD-3 in 4-cell stage embryos and these two proteins remained asymmetric at this stage with EMS and P2 having significantly less protein than the AB cell lineage (Fig 4E and 4F). SLD-2 abundance was also significantly lower in the P2 cell than the EMS cell (Fig 4F). Together these data show that SLD-2 and SLD-3 are present at levels that inversely correlate with S-phase length in the 2- and 4-cell *C*. *elegans* embryo.

### PAR proteins control SLD-2 asymmetry

**Fig 5:**
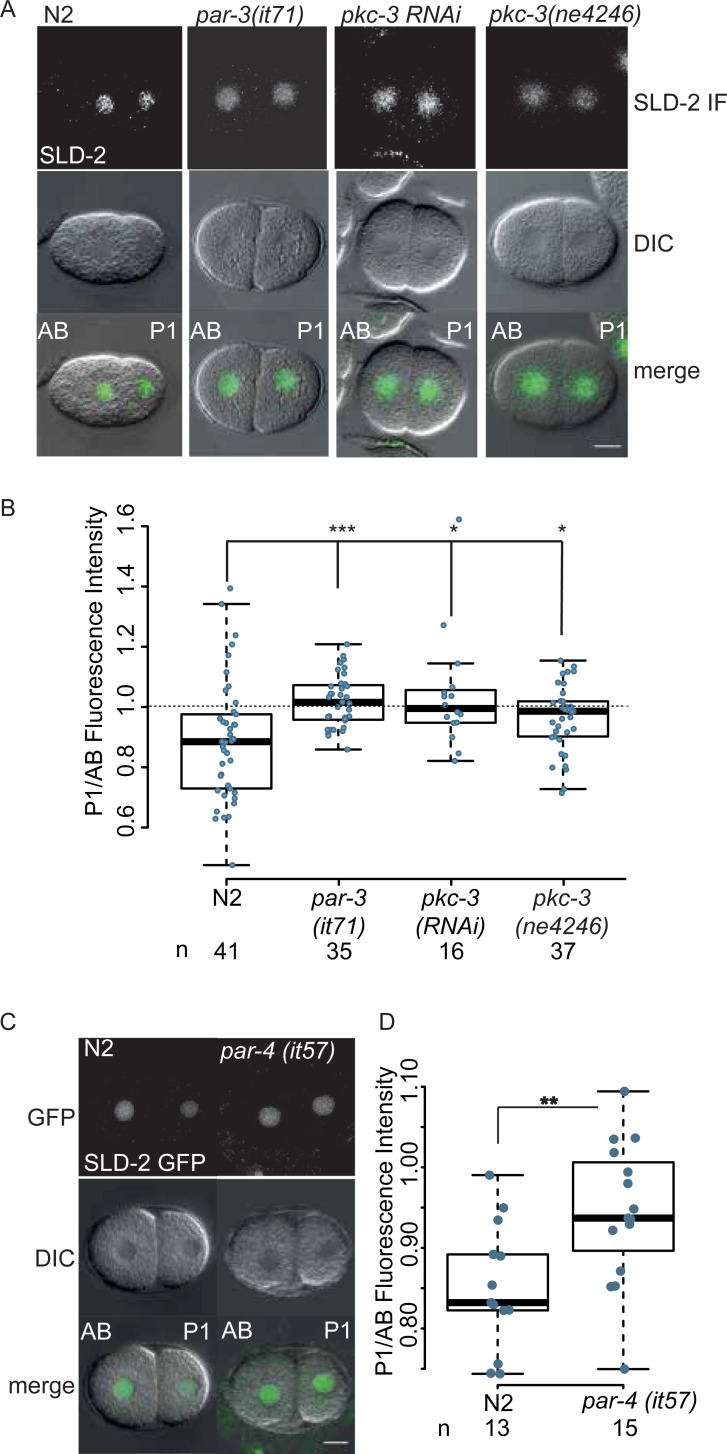
SLD-2 asymmetry is polarity-dependent A. Images of two-cell embryos from the indicated strains after SLD-2 IF. Scale bar is 10 μm. N2, *par-3(it71)* and *pkc-3 RNAi* animals were grown at 20°C. The temperature-sensitive strain *pkc-3(ne4246)* was grown at 25°C **B.** Box plot of the data from A. p-values were obtained using the Wilcoxon rank sum test. **C.** Images of two-cell embryo of wild type (N2) and the temperature-sensitive *par-4(it57)* mutant containing *mex-5p*::*sld-2-gfp*::*tbb-2 3UTR* grown at 25°C. The fluorescent GFP signal was detected with Laser Confocal Scanning Microscopy. Scale bar is 10 μm. **D.** Box plot of the data from C. p-values were obtained using the Wilcoxon rank sum test.

The PAR polarity proteins (PAR-1 to -6) and PKC-3, which specify the anterior-posterior (A–P) axis in the early embryo, also regulate the asynchrony of cell division between the AB and P1 blastomeres [2]. *par-3* and *pkc-3* mutants divide synchronously and symmetrically at the two-cell stage [2,33] and significantly loss of function of either of these polarity genes resulted in subsequent symmetrical localisation of SLD-2 in the AB and P1 cell (Fig 5A and 5B).

We have previously shown in *Xenopus* that nuclear-to-cytoplasmic ratios can affect S-phase length due to the amount of limiting replication initiation factors inherited after cell division [13]. As *par-3* and *pkc-3* mutants divide symmetrically (Fig 5A), we wondered whether the subsequent symmetry of SLD-2 was simply a consequence of equal distribution of cellular content after division. To test this we analysed the distribution of SLD-2 in *par-4* mutant embryos, which divide synchronously but still asymmetrically at the two-cell stage, resulting in AB/P1 cells of similar size to wild type [34]. Significantly, SLD-2 was symmetrically localised in *par-4* mutant embryos, even though the P1 cell is smaller than the AB cell in these mutants (Fig 5C and 5D). Together this suggests that SLD-2 localisation is actively regulated by the PAR protein network not simply by the cellular volume at division.

### PKC-3 interacts with SLD-2 and causes SLD-2 asymmetry in the embryo

**Fig 6:**
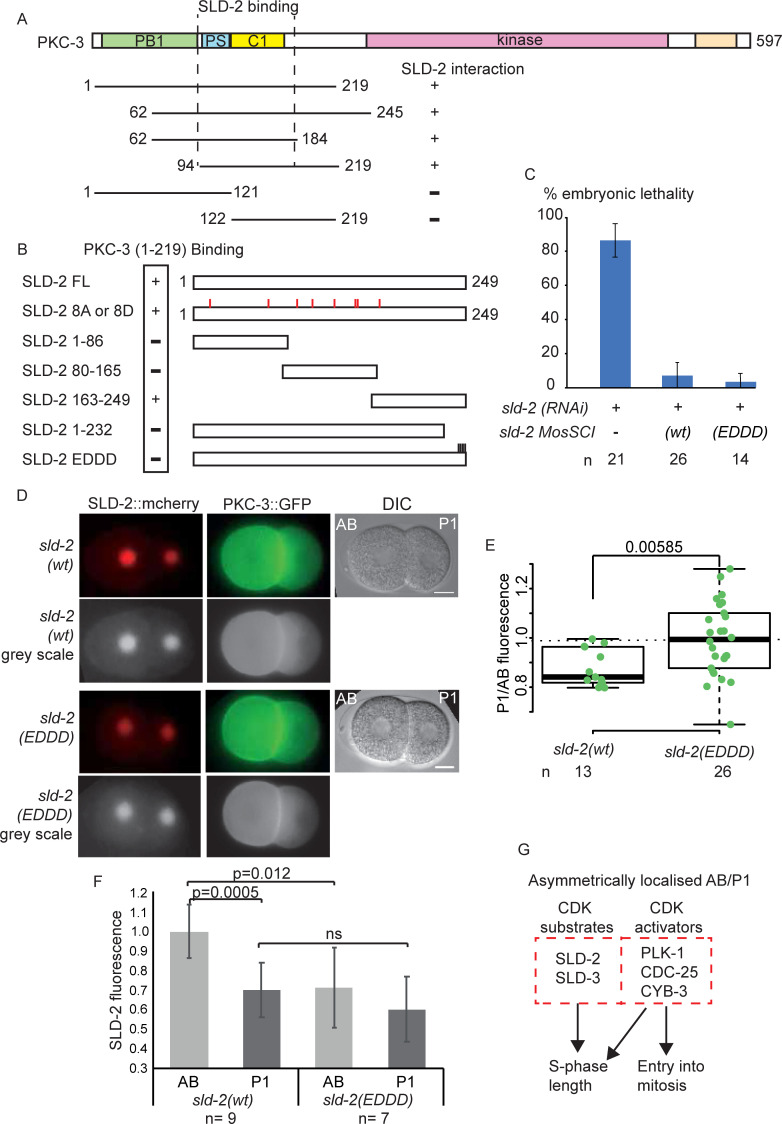
SLD-2 asymmetry is PKC-3 interaction-dependent A. (Top) Scale diagram of *C*. *elegans* PKC-3. (Bottom) Diagram of PKC-3 fragments that were tested for yeast two-hybrid interaction with full length SLD-2. +/- represents whether the interaction was positive or negative. **B.** Scale diagram of fragments of SLD-2 that were tested for yeast two-hybrid interaction with PKC-3 (1–219). +/- represents whether the interaction was positive or negative. SLD-2 8A or 8D refers to all 8 CDK sites mutated either alanine or aspartic acid. SLD-2 EDDD refers to residues 246–249 (KKKY) mutated to EDDD. **C**. Embryonic lethality 16-40hrs post-injection from the wild type strain (N2) or MosSCI generated *mex-5p*:::*sld-2(WT)*::*gfp*::*tbb-2 3UTR* or *mex-5p*::*sld-2(EDDD)*::*gfp*::*tbb-2 3UTR* strains. SLD-2 RNAi was done by injection. Error bars are 95% CI. **D.** Live imaging of PKC-3 GFP and SLD-2 mCherry or SLD-2 EDDD mCherry generated by CRISPR. Scale bar is 10 μm. **E.** Detection of the nuclear SLD-2 signal by IF. Box plot graph shows the ratio of the fluorescent signal intensity from the P1 cell over the signal from the AB cell from the wild type strain (N2) and the mutant *sld-2 EDDD* generated by CRISPR. **F**. SLD-2 GFP signal measurements in the AB and P1 cells. The GFP signal was normalized to the cytoplasmatic signal in the AB cell and the average signal of the AB nucleus of the *sld-2(wt)* was set to 1. Error bars are 95% CI. **G**. Both CDK substrates (SLD-2 and SLD-3) and CDK activators (PLK-1, CDC-25 and CYB-3) are asymmetrically localised at the two-cell stage in *C*. *elegans*. The relative contribution of these localisations to asynchronous embryonic cell cycle lengths remains to be determined.

To understand the molecular mechanism of SLD-2 asymmetry, we performed a yeast two-hybrid screen between SLD-2 and a cDNA library from *C*. *elegans* embryos. One of the hits from this screen was the polarity factor *pkc-3*, which is essential for defining the anterior domain in the one-cell embryo [35]. SLD-2 interacts with the PKC-3 region 94–184, which encompasses the pseudosubstrate (PS) and C1 domains (Fig 6A). To assess the function of this interaction *in vivo*, we set out to identify a separation of function mutant in *sld-2*, which lacked the PKC-3 interaction. Using yeast two-hybrid analysis we narrowed down the interaction to the very C-terminus of SLD-2, region 232–249 (Fig 6B). This is a highly basic region of SLD-2 (S4A Fig), which lacks any CDK sites. Indeed the SLD-2 mutant lacking all 8 CDK sites (either mutated to alanine or aspartic acid, 8A/8D) still interacted with PKC-3 (Fig 6B). To identify a mutant that no longer interacted with PKC-3 we made scanning mutations in the region 232–249 (S4B and S4C Fig). A mutation that converted the very C-terminal 4 amino acids from KKKY to the acidic residues EDDD indeed resulted in loss of the interaction with PKC-3 (Fig 6B and S4B and S4C Fig) and we hereafter refer to this mutant as *sld-2(EDDD)*. To check whether these mutations affect the essential functions of *sld-2*, we tested whether *sld-2(EDDD)* expression rescued the lethality of *sld-2* RNAi. Insertion of either *sld-2* wild type or the *EDDD* mutant at a MosSCI site fully rescued the lethality of *sld-2* RNAi (Fig 6C) strongly suggesting that the *sld-2(EDDD)* mutant is not defective in any of the essential functions of *sld-2*.

To investigate the significance of the SLD-2 interaction with PKC-3 for SLD-2 localisation we generated *sld-2(WT)* and *sld-2(EDDD)* alleles by CRISPR. Homozygous *sld-2(EDDD)* strains were viable and showed no sterility phenotypes as expected from the MosSCI strains (Fig 6C). Importantly while the wild type SLD-2 showed asymmetric localisation in the AB cell versus the P1 cell in two-cell embryos as expected, the *sld-2(EDDD)* mutant which can no longer interact with PKC-3 exhibited equal localisation in AB and P1 cells (Fig 6D and 6E). This suggested that the interaction of SLD-2 with PKC-3 is important for the asymmetric localisation of SLD-2 in the early *C*. *elegans* embryo. Although PKC-3 is largely cytoplasmic and SLD-2 is mostly nuclear, SLD-2 becomes entirely cytoplasmic upon nuclear envelope breakdown and we do observe an enrichment of both nuclear and cytoplasmic SLD-2, in the AB versus the P1 cell (S4D Fig). Quantification of the mcherry signal showed that the *sld-2(EDDD)* mutant is less abundant in the AB nucleus than the wild type protein (Fig 6D and 6F), suggesting that loss of interaction with PKC-3 results in levels of SLD-2 protein that are equivalent to the P1 nucleus.

Having identified a mutant of *sld-2* that is no longer asymmetrically localised in two-cell embryos, we wondered if this had an effect on the cell cycle duration of this stage. For example if SLD-2 protein is limiting for S-phase length, then less protein in the AB nucleus might result in the AB cell of the *sld-2(EDDD)* mutant having a shorter cell cycle. Despite this the *sld-2(EDDD)* mutant alone had no effect on the duration of the AB or P1 cycle length (S5 Fig). Together these data show that the PAR protein network controls SLD-2 asymmetry through PKC-3 interaction, but on it’s own symmetrical localisation of SLD-2 is not sufficient to alter the cell cycle dynamics at the two-cell stage.

## Discussion

It is vital for all organisms to make a perfect copy of the genome in every cell division. For eukaryotes this is achieved in large part by linking DNA replication control to CDK activation at the G1-S transition [7]. CDK plays a vital dual role in DNA replication, both as an inhibitor of the helicase loading step in the initiation reaction (a process called licensing) and as an activator of these loaded helicases during replisome assembly. In budding yeast, CDK activates replisome assembly by phosphorylation of Sld2 and Sld3, but the relative contribution of phosphorylation of these two proteins to replication initiation differs in other species [17]. CDK phosphorylation of the metazoan orthologue of Sld3 (Treslin/Ticrr/C15orf42) has been shown to be important for S-phase progression in human cells in culture [21,25,28], but evidence for an essential role for Sld2 (RecQ4/RecQL4) phosphorylation in vertebrate cells is lacking. Conversely, CDK phosphorylation of Sld3 is not essential in the fission yeast *S*. *pombe* and Sld3 orthologues are so far absent in *D*. *melanogaster* [17].

By characterising *sld-2* and *sld-3* in the nematode *C*. *elegans*, we show for the first time outside of budding yeast that both of these proteins mediate essential interactions with MUS-101 (Dpb11/Cut5/TopBP1) through critical CDK sites (Figs 1 and 2 and [18]). Importantly phospho-mimicking mutants in both *sld-2* and *sld-3* that drive interactions with MUS-101 are partially sufficient for cyclin E function in *C*. *elegans* (Fig 3). As the rescue of the *cye-1* RNAi with *sld-2* and *sld-3* bypass mutants is only partial we cannot rule out that there may be other CDK targets required for replication initiation in *C*. *elegans*, although it is also the case that the D and E mutants of *sld-2/sld-3* are not perfect phospho-mimics (Fig 2E). In addition, Cyclin E has multiple functions in *C*. *elegans* such as contributing to embryo polarity [36] and cell cycle re-entry of differentiated cells [31,37]. It is also important to note that since most cells differentiate and become post-mitotic before the completion of embryonic development in *C*. *elegans* [32], the viability assays used in this study only assess the contribution of Cyclin E to cell proliferation during early embryogenesis.

The early embryonic divisions in many metazoa, such as in *Drosophila*, zebrafish and *Xenopus*, are extremely rapid, lack gap phases and are characterised by high rates of replication initiation. Cell cycle lengthening in these embryonic divisions coincides with activation of DNA damage checkpoint kinases and the down regulation of cyclin-dependent kinase (CDK) activity, through the inhibitory phosphorylation of CDK by Wee1 and down-regulation of the counteracting phosphatase Cdc25 (String/Twine in *Drosophila*) [38,39]. Inhibitory phosphorylation of CDK is likely critical for the introduction of G2 phase and for delaying entry into mitosis. In *Drosophila* however increasing CDK activity can also reduce S-phase length at the mid-blastula transition (MBT) [14], although expression of CDK mutants that cannot be inhibited by Wee1 does not affect S-phase length at the MBT in *Xenopus* or zebrafish [15,16].

In *C*. *elegans*, CDC-25 and the Polo-like kinase PLK-1 (which increases the nuclear accumulation of CDC-25) preferentially localise to the faster dividing AB cell in the early embryo [2,4–6], while checkpoint activation has been proposed to preferentially occur in the P1 cell [40]. RNAi of *wee-1* in *C*. *elegans* indeed results in faster division of the P1 cell [6]. Therefore in both *Drosophila* and *C*. *elegans*, inhibitory phosphorylation of CDK plays an important role in cell cycle lengthening in the embryo. Despite this, in both of these organisms cell cycle elongation begins with changes in replication initiation [41,42], but how this is achieved is not clear. In *Drosophila* embryos, CDK activity prevents the chromatin binding of Rif1, and loss of Rif1 to a large extent prevents normal cell cycle elongation in cycle 14 [43]. Rif1 is known to inhibit replication initiation through counteraction of Dbf4-dependent kinase (DDK), but also causes changes in chromatin structure [44]. Despite this, RNAi of Rif1 is not sufficient to accelerate the early embryonic divisions in *C*. *elegans* (S5B Fig).

Here we show that bypass of SLD-2 and SLD-3 activation by CDKs is not sufficient to change the cell cycle length in the early embryo (Fig 4A). This suggests that CDK phosphorylation of these two replication substrates is not limiting for S-phase length at least at the two-cell stage. Instead we show that the SLD-2 and SLD-3 proteins themselves are asymmetrically distributed (Figs 4–6). It is striking that both the regulators and the substrates of CDKs are asymmetrically localised in the AB versus P1 cell in *C*. *elegans* (Fig 6G and [2,4–6]). Although symmetric localisation of SLD-2 alone was not sufficient for alter the early embryonic divisions (S5 Fig), we do not currently know how SLD-3 asymmetry is controlled to test the effect of equal distribution of both proteins towards cell cycle length.

SLD-2 asymmetry in the *C*. *elegans* embryo is controlled by direct interaction with the polarity factor PKC-3 (Fig 6), which is preferentially localised at the anterior of the embryo [33,45]. A possible mechanistic explanation for SLD-2 accumulation in the anterior AB nucleus over the posterior P1 nucleus is therefore that SLD-2 becomes enriched in the AB cytoplasm (S4D Fig) by virtue of the established asymmetry of PKC-3. In line with this hypothesis, the localisation of both SLD-2 and the anterior polarity proteins, including PKC-3 are dependent on *par-3* and *par-4* (Fig 4 and [46,47]). It is also possible that PKC-3 interaction with SLD-2 may preferentially stabilise SLD-2 in the AB cell or even regulate SLD-2 by direct phosphorylation. It is intriguing that the asymmetric distribution of SLD-2 is first detected after cytokinesis forms the two-cell embryo (S3D Fig).

Although cell polarity has been shown to be required for S-phase length control in the early *C*. *elegans* embryo [41], to our knowledge we have provided the first direct link between the polarity network proteins and factors that are essential for DNA replication initiation. This study may provide a platform to understand the mechanism by which programmed developmental cues directly influence S-phase length. As the human *pkc-3* orthologues are frequently mutated in cancers [48], this new link between atypical PKC and factors required for genome duplication may provide a novel mechanism by which this tumour suppressor affects cell proliferation.

## Materials and Methods

### Strains

Standard conditions were used to maintain *C*. *elegans* cultures (Brenner, 1974). The *C*. *elegans* Bristol strain N2 was used as wild type strain. Strains created by MosSCI contain codon altered versions of *sld-2* or *sld-3* to make them refractory to RNAi of endogenous *sld-2/sld-3*. The following strains were used in this study: JA1564 (*weSi35 [Pmex-5*::*sld-2(wt)*::*egfp/tbb-2 3’UTR; cb-unc-119(+)]* II; *cb-unc-119 (ed9) III)*, JA1563*(weSi34 [Pmex-5*::*sld-2(8D)*::*egfp/tbb-2 3’UTR; cb-unc-119(+)]* II; *cb-unc-119(ed9)* III) [18], KK300 *(par-4(it57*ts*)V)* [34], KK571 *(lon-1(e185) par-3(it71)/qC1 [dpy-19(e1259) glp-1(q339)] III)* [49], KK1228 *(pkc-3(it309 [gfp*::*pkc-3])* II), WM150 *(pkc-3(ne4246)*II*)* [33] and EU548 (*div-1(or148*ts*)* III) [24].

Strains introduced in this study.

**Table:** 

name	PAZ and zap name
*sld-3 WT*	PAZ1 *(zapSi1 (unc-119(ed9)* III; *[Pmex-5*::*sld-3(wt)*:: *mcherry/tbb-2 3’UTR; cb-unc-119(+)]* IV)
*sld-3 2A*	PAZ2 *(zapSi2 (unc-119(ed9)* III; *[Pmex-5*::*sld-3(2A)*:: *mcherry/tbb-2 3’UTR; cb-unc-119(+)]* IV)
*sld-3 2D*	PAZ3 *(zapSi3 (unc-119(ed9)* III; *[Pmex-5*::*sld-3(2D)*:: *mcherry/tbb-2 3’UTR; cb-unc-119(+)]* IV)
*sld-3 2E*	PAZ4 *(zapSi4 (unc-119(ed9)* III; *[Pmex-5*::*sld-3(2E)*:: *mcherry/tbb-2 3’UTR; cb-unc-119(+)]* IV)
*sld-3 T487A*	PAZ5 *(sld-3 (zap5[sld-3 (T487A)]))*
*sld-3 T487D*	PAZ6 *(sld-3 (zap6[sld-3 (T487D)]))*
*sld-3 T487E*	PAZ7 *(sld-3 (zap7[sld-3 (T487E)]))*
*sld-3 T438A*	PAZ8 *(sld-3 (zap8[sld-3 (T438A)]))*
*sld-3 T438D*	PAZ9 *(sld-3 (zap9[sld-3 (T438D)]))*
*sld-3 T438E*	PAZ10 *(sld-3 (zap10[sld-3 (T438E)]))*
*+/sld-3 2E*	PAZ11 *(tmC18 [dpy-5(tmIs1200)] I; +/ sld-3(zap11[sld-3(2E)])*
*+/sld-3 2D*	PAZ12 *(tmC18 [dpy-5(tmIs1200)] I; +/ sld-3 (zap12[sld-3 (2D)])*
*sld-2 EDDD*	PAZ13 *(sld-2(zap13[sld-2*^*(EDDD*^*]))*
*sld-2 EDDD-GFP*	PAZ14 *(zapSi14 (unc-119(ed9))* III; *[Pmex-5*::*sld-2*^*(EDDD)*^:: *gfp/tbb-2 3’UTR; cb-unc-119(+)])* IV)
*sld-2 EDDD 3xFLAG mCHERRY*	PAZ15 *(sld-2(zap15[sld-2(EDDD)*::*3xFLAG*::*mcherry]))*
*sld-2 wt 3xFLAG mCHERRY*	PAZ16 *(sld-2 (zap16[sld-2(+)*::*3xFLAG*::*mcherry]))*
*sld-2 8D; sld-3 2E*	PAZ17 *(weSi34; zapSi4)*
*sld-2 8D; sld-3 2D*	PAZ18 *(weSi34; zapSi3)*
*sld-2 8D; sld-3 2E*	PAZ19 *(weSi34; +/zap11)*
*sld-2 wt; sld-3 wt*	PAZ20 *(weSi35; zapSi1)*
*sld-2 wt; sld-3 2D*	PAZ21 *(weSi35; zapSi3)*
*sld-2 8D; sld-3 wt*	PAZ22 *(weSi34; zapSi1)*

### Homology search

Identification of ZK484.4 as a potential Sld3 orthologue was using (Position-Specific Iterated BLAST (PSI-BLAST NCBI), using the yeast Sld3 Treslin domain as the input.

### Yeast-Two-Hybrid assays

Performed as previously described [18].

### Immunostaining

SLD-2 Immunofluorescence: Young adults were cut on a slide in a drop of M9 to release the young embryos. Embryos were freeze cracked and fixation with additional antibody incubation and washing steps were performed as described in [18]. The primary antibody was rabbit anti-SLD-2 (Ab 5058; [18]). SLD-2 antibody was used in a dilution of 1:100. Secondary antibody labelled with AlexaFluor488 anti-rabbit were obtained from Molecular Probes and used in a dilution of 1:500. Hoechst stain was added 1:1000 into the secondary antibody dilution. Vectashield Antifading Mounting Media was used for mounting. The temperature-sensitive mutant *pkc-3(ne4246)* was kept at 25°C overnight before the immunofluorescence experiment.

SLD-3 mCHERRY Immunofluorescence: Immunofluorescence was performed using a protocol adapted from [18]. Young adult hermaphrodites were cut to release embryos onto 0.1% poly-lysine (Sigma, P8920)-coated slides. Slides were covered with a 22x50mm coverslip and frozen on dry ice for 20 minutes. The coverslip was quickly removed while the slide where still frozen to permeabilise the embryos. Embryos were fixed in ice cold methanol for 30 seconds. Slides were additionally fixed in a fixing solution containing 4% Paraformaldehyde, 80mM Hepes, 1.6mM MgSO4 and 0.8mM EGTA in PBS for 20 minutes at room temperature (RT). Samples were then washed in PBS and 0.2% Tween 20 (PBST) five times over 30 minutes, followed by blocking in 1% BSA in PBST (PBSTB) for one hour at RT. Slides were incubated in the primary antibody rabbit anti-RFP (600-401-379; Rockland antibodies and assays) (1:200) PBSTB solution overnight at 4°C. Slides were washed in PBST five times over 30 minutes, followed by incubation with the secondary antibody Alexa Fluor 594-conjugated donkey anti-rabbit antibody (A-21207; Molecular Probes) (1:500) and Hoechst 33342 stain (1:1000 final concentration 1 μg/mL) in PBSTB at RT for one hour. Samples were washed in PBST five times over 30 minutes and PBS five times over 20 minutes. Vectashield Antifading Mounting Media was used for mounting.

### RNAi by feeding

The temperature-sensitive mutant strain EU548 (*div-1(or148*)) was synchronized by bleaching and grown at 15°C together with N2 wild type control. RNAi inducing plates were spotted with *sld-3* RNAi (this study, ZK484.4 ORF was cloned into L4440 plasmid) bacteria grown at 37°C for 7hrs. RNAi bacteria grown at 37°C for 7hrs. L1 worms were seeded on RNAi inducing plates and kept at 21°C until adulthood. Young adults were singled on separate NGM plates and the embryonic lethality of their progeny was determined.

For the embryonic lethality of *cdk-2*, *cyb-1* and *cye-1* RNAi bacteria were grown at 37°C for 7hrs in LB containing Ampicillin. Worm strains were synchronized by bleaching. L4 worms were seeded on RNAi inducing plates with the respective RNAi bacteria until they reached adulthood and were allowed to lay eggs for 24hrs. Plates were kept at 25°C. The percentage of embryonic lethality of the F1 generation was calculated by counting the number of hatched and unhatched progeny.

The wildtype strain N2 was used for *pkc-3* RNAi. Worms were synchronized by bleaching. Plates were kept at 20°C. Mid L3 animals were seeded on RNAi inducing plates. Young adults were used for SLD-2 immunofluorescence staining. A subset of worms was singled to confirm PKC-3 knockdown by assessing embryonic lethality.

*cyb-3* RNAi bacteria were grown in 37°C for 7hrs in LB containing Ampicillin. 2.5% (v/v) *cyb-3* bacteria diluted in L4440 control bacteria was used for seeding RNAi inducing plates. Worms were synchronized by bleaching. Plates were kept at 20°C. L3 larva were seeded on RNAi plates. Young adult worms were singled out and allowed to lay eggs for 24hrs. After additional 36hrs the embryonic lethality was determined by counting the hatched and the unhatched progeny.

All RNAi experiments included the feeding of L4440 bacteria as a control.

### RNAi by injection

*sld-3* RNAi injections: N2, PAZ1, PAZ2, PAZ3 and PAZ4 young adult hermaphrodites were injected with *sld-3* double stranded RNA, containing the entire coding region of ZK484.4 with a concentration of 100ng/μl. Injected worms were kept at 20°C and singled to separate NGM plates to assess the embryonic lethality in the F1 generation.

*sld-2* RNAi injections: N2, JA1564 and PAZ14 were synchronized by bleaching and grown at 20°C to the young adult stage. Young hermaphrodites were injected with *sld-2* double stranded RNA prepared from T12F5.1 with a concentration of 150ng/μl. Injected worms were incubated at 20°C. The injected worms were singled out after 16hrs post-injection and transferred on new plates every 24hrs for 3 days. The plates were assessed for total egg production and lethality in the embryos.

### Microscopy and image analysis

Immunofluorescence of SLD-3 mCHERRY was visualized on a DeltaVision widefield microscope (ImSol/GEHealthcare) with a 100x/1.4 NA oil immersion objective. Z-stack images were deconvolved using default settings in the SoftWorx software version 5.5.0 Release 6 using experimental PSFs.

The fluorescent signal of the nuclei was analysed with ImageJ. Z-stack images were combined using the Z-stack tool for maximum projection. The signals of the AB and P1 nuclei were normalized against background and the signal ratio of P1/AB visualized using R.

The immunofluorescent signal of SLD-2 staining and in-vivo imaging of the SLD-2 GFP signal in JA1563 and *weSi35; par-4(it57)* were visualized using Leica SP8 confocal using a 63x/1.4NA oil immersion objective. Z-stack images through the whole embryo were taken. Images were analysed with ImageJ. Using the maximum projection z-stack tool the fluorescent signal of the nuclei was measured and normalized for background signal.

Relative Fluorescence analysis of SLD-3 and SLD-2 signal in the four-cell embryo:

Z-stack images of SLD-3 and SLD-2 IF was analysed using ImageJ. The fluorescent intensity signal of the different nuclei was obtained using the maximum projection tool. The signal in ABa was set to 1. The signal was normalized for background using the signal of the AB cytoplasm.

### SLD-2 GFP localization kinetics

Time lapse z-stack movies of 1 min intervals were taken from early embryos of JA1564 with a DeltaVision widefield microscope (ImSol/GEHealthcare) using a 60x oil objective. The embryonic development of the pronuclei meeting to the nuclear envelope breakdown of the AB cell was recorded. Image analysis was carried out using a custom script (https://github.com/gurdon-institute/Two_Cells/blob/master/Two_Cells.py) for Fiji. The script extracts the best-focused z-slice from each frame of a 5D hyperstack of the two-cell embryo from the first division to nuclear envelope breakdown prior to the second division. The two cells are mapped using smoothed local standard deviation of intensity in the DIC channel, and a mask is generated by Otsu thresholding. A standard watershed was initially applied to segment the two cells, and where this was unsuccessful due to lack of concave features the cells were divided approximately using the minor axis of a fitted ellipse. Nuclei were segmented from the maximum fluorescence intensity Z-projection of the GFP signal in channel 1 using a difference of Gaussians filter and Kapur's Maximum entropy threshold. This segmentation allows nuclear and normalised, background-corrected cytoplasmic signal intensity to be measured over time.

### Cell cycle length analysis

Cell cycle length was analysed for N2 and *weSi34; zapSi4*. Cell cycle length analysis was also done for N2 and *sld-2(zap13[sld-2EDDD])* for *wee1*.*3* RNAi. Time lapse z-stack movies were taken starting from pronuclei migration until the four-cell stage of the early embryo development in *C*. *elegans*. The time lapse interval was 8s. Cell cycle time of the AB cell was calculated as the time starting from pronuclei fusion until nuclear envelope breakdown of the AB cell. Movie was taken using a DeltaVision widefield microscope (ImSol/GEHealthcare) with Nomarski optics and a 60X oil objective. Cell cycle timing of the P1 cell was calculated as the time from pronuclei fusion until the nuclear envelope breakdown of the P1 cell.

### Progeny assays

N2, JA1563, PAZ3, *weSi34; zapSi3* and *weSi34; zapSi4* were synchronized by bleaching. Plates were kept at 25°C. L4 (P0) were singled on NGM plates seeded with OP50. After 24hrs P0 worm was transferred on new plate. This was done for five days until egg production stopped. The total production of fertilized eggs from each animal was calculated.

CRISPR *sld-3(2D)* and *sld-3(2E)* progeny analysis:

Heterozygous hermaphrodites of *sld-3(2D)* and *sld-3(2E*) and homozygotes single site mutants were allowed to lay eggs at 20°C. Their larvae were singled on new plates and checked for sterility. After two days after reaching adulthood they were lysed and genotyped by PCR for the *sld-3* locus.

### Embryonic lethality assays

The *sld-3* Crispr mutants (*sld-3(paz5)*, *sld-3(paz6)*, *sld-3(paz7)*, *sld-3(paz8)*, *sld-3(paz9)*, *sld-3(paz10)*) were tested for embryonic lethality. N2 was used as a control. Plates were kept at 20°C. Young adults were singled on new plates and allowed to lay eggs for 24hrs. The hermaphrodite was then removed and the embryonic lethality was determined after additional 36 hours.

The *sld-2(8D); sld-3(2E)* MosSCI strain was tested for embryonic lethality. N2 and weSi34; zapSi4 were synchronized by bleaching. The plates were grown in 25°C until mid-J4 stage. Worms were singled on new plates and transferred again to new plates every 24hrs for five days until egg production stopped. Living larva and unhatched eggs were counted after additional 24hrs. Experiment was performed in 25°C.

### gRNA and oligo list

**Table:** 

Oligonucleotide list:	Oligonucleotide name	Sequence 5’-3’
Genotyping zapSi14	PZ2678 PZ2092	TCGGATCAGAATTTCCAAAAGA TGAGACTTTTTTCTTGGCGG
Crispr *zap13*	guide target sequence repair template	CAACUUUCGCUUCUGCUCUG AAAAATGAAATTCTAGACAAATTA GAATATTCAGTCATCATCCTCAAACATCTGCTTGCGCTTGAGCTTGCGCTTTTGTTCGGCAGTAACTTTTCCGCGAACGAATTGCTTCTTTCTCA
Crispr *zap15*	guide target sequence repair template	CAACUUUCGCUUCUGCUCUG 5’ CTGAGAAAGAAGCAATTCGTTCGCGGAAAAGTTACTGCCGAACAAAAGCGTAAGCTCAAGCGCAAGCAGATGTTTGAGGATGATGACGAGGCTGGAGAAGAGGGAGAAAAGgactacaaGgaccaCgacggAgattaCaaGgatcaCgaTatcgattacaaggatgacgatgacaagATGGTCTCAAAGGGTGAAGAAGATAACATGGCAATTATTAAAGAGTTTATGCGTTTCAAGGTGCATATGGAGGGATCTGTCAATGGGCATGAGTTTGAAATTGAAGGTGAAGGAGAAGGCCGACCATATGAGGGAACACAAACCGCAAAACTAAAGGTAACTAAAGGCGGACCATTACCATTCGCCTGGGACATCCTCTCTCCACAGTTCATGTATGGAAGTAAAGCTTATGTTAAACATCCGGCAGATATACCAGATTATTTGAAACTTTCATTCCCGGAGGGTTTTAAGTGGGAACGCGTAATGAATTTTGAAGACGGAGGAGTTGTTACAGTGACGCAAGACTCAAGCCTCCAAGATGGAGAATTTATTTATAAAGTCAAACTTCGAGGAACGAATTTCCCCTCGGATGGACCTGTTATGCAGAAGAAGACTATGGGATGGGAAGCTTCAAGTGAAAGAATGTACCCTGAAGACGGTGCTCTTAAGGGAGAGATTAAACAACGTCTTAAATTGAAAGATGGAGGACATTACGATGCTGAGGTGAAGACAACTTACAAAGCCAAAAAACCAGTTCAGCTGCCAGGAGCGTACAATGTTAATATTAAACTGGATATCACCTCCCACAACGAGGATTACACTATCGTTGAGCAATATGAAAGAGCTGAAGGGCGGCACTCGACAGGTGGCATGGATGAATTGTATAAGtagATATTCTAATTTGTCTAGAATTTCATTTTTAC3’
Crispr *zap16*	guide target sequence repair template	CAACUUUCGCUUCUGCUCUG 5’ CTGAGAAAGAAGCAATTCGTTCGCGGAAAAGTTACTGCCGAACAAAAGCGTAAGCTCAAGCGCAAGCAGATGTTTAAGAAAAAGTATGAGGCTGGAGAAGAGGGAGAAAAGgactacaaGgaccaCgacggAgattaCaaGgatcaCgaTatcgattacaaggatgacgatgacaagATGGTCTCAAAGGGTGAAGAAGATAACATGGCAATTATTAAAGAGTTTATGCGTTTCAAGGTGCATATGGAGGGATCTGTCAATGGGCATGAGTTTGAAATTGAAGGTGAAGGAGAAGGCCGACCATATGAGGGAACACAAACCGCAAAACTAAAGGTAACTAAAGGCGGACCATTACCATTCGCCTGGGACATCCTCTCTCCACAGTTCATGTATGGAAGTAAAGCTTATGTTAAACATCCGGCAGATATACCAGATTATTTGAAACTTTCATTCCCGGAGGGTTTTAAGTGGGAACGCGTAATGAATTTTGAAGACGGAGGAGTTGTTACAGTGACGCAAGACTCAAGCCTCCAAGATGGAGAATTTATTTATAAAGTCAAACTTCGAGGAACGAATTTCCCCTCGGATGGACCTGTTATGCAGAAGAAGACTATGGGATGGGAAGCTTCAAGTGAAAGAATGTACCCTGAAGACGGTGCTCTTAAGGGAGAGATTAAACAACGTCTTAAATTGAAAGATGGAGGACATTACGATGCTGAGGTGAAGACAACTTACAAAGCCAAAAAACCAGTTCAGCTGCCAGGAGCGTACAATGTTAATATTAAACTGGATATCACCTCCCACAACGAGGATTACACTATCGTTGAGCAATATGAAAGAGCTGAAGGGCGGCACTCGACAGGTGGCATGGATGAATTGTATAAGtagATATTCTAATTTGTCTAGAATTTCATTTTTAC3’
Genotyping *zap13*, *zap15 and zap16* Followed by SmlI digest	PZ2957 PZ2958	TTCAGGATCTCAAAATGCTTCTCTTACC AGGCGCACACTTATTTGCAATAATTAAA
*sld-3* T438 Mutation	Guide	ctacaagcatttttttgtcg
	Repair template T438A	acaacacgaacaagtcgatctgactacaagcatttCttCgtTgaAgacGCTccagatgagaaatatcagaagaaaacgacgaaagt
	Repair template T438D	acaacacgaacaagtcgatctgactacaagcatttCttCgtTgaAgacGACccagatgagaaatatcagaagaaaacgacgaaagt
	Repair template T438E	acaacacgaacaagtcgatctgactacaagcatttCttCgtTgaAgacGAGccagatgagaaatatcagaagaaaacgacgaaagt
*sld-3* T487 Mutation	guide	agaagtcaaacagacaccaa
	Repair template T487A	aaagaagaaattgatgaagaagtcaaacaAGcTccGatggcaaagctgagagcatcagcgaaActtaat
	Repair template T487D	aaaagaagaaattgatgaagaagtcaaacaAGACccGatggcaaagctgagagcatcagcgaaActtaat
	Repair template T487E	aaaagaagaaattgatgaagaagtcaaacaAGAGccGatggcaaagctgagagcatcagcgaaActtaat

### CRISPR

CRISPR strains were generated according to [50]. Reagents were purchased at IDT. For injections the N2 wildtype strain was used. Injection mix contained the *dpy-10* marker to identify jackpot broods. 10μl injection mix was prepared. TracrRNA (360μM) 0.24μl, dpy10 RNA (100μM) 0.32μl, target guide RNA (200μM) 0.24μl, duplex buffer 0.5μl and 3.7μl water were mixed and incubated at 95C for 5min. Mix was put at RT for 5min to cool down. 0.25μl Cas9 was added and waited for another 5min before the rest was added to the final mix. 0.2μl dpy-10 repair template (500ng/μl), 0.44μM of repair template and water were added to a total of 10μl. N2 were kept at 20C post-injection and transferred to new plates after 12hrs for egg laying. P0 were again transferred to new plates after 24hrs. F1 were singled out from jackpot brood consisting of about 30–40% dpys or rollers. After egg laying F1 was tested for mutation by single-worm lysis followed by PCR. For the identification of *zap13*, *zap15* and *zap16* oligonucleotides in the Table above were used. The identification of the *zap13* allele additionally required SmlI digest of the PCR product. Independent homozygous lines were isolated and outcrossed twice with N2 before the mutant allele was once again confirmed by Sanger sequencing.

## Supporting information

S1 Fig Analysis of single CDK site mutants in *sld-3*. **A**. Alignment of the CDK sites in Sld3/Treslin required for the interactions with Dpb11/TopBP1. The amino acid numbers of the two orthologous CDK sites in *C*. *elegans* SLD-3 are indicated below. **B** and **C**. Box plots of progeny size (B) and embryonic lethality (C) of a wild type strain (N2) or strains with the CDK site T487 mutated to alanine, aspartic acid or glutamic acid by CRISPR. **D** and **E**. As B/C except for the T438 CDK site. **F.** Table of orthologue names for Sld3, Sld2 and Dpb11
(EPS)

S2 Fig Bypass of Sld3 and Sld2 phosphorylation is partially sufficient for cyclin E function. **A.** Box plots of embryonic lethality from strains containing extra copies of *sld-2* / *sld-3*, both of which are inserted at a MosSCI sites and expressed from the *mex-5* promoter. n refers to the number of mothers analysed. **B.** Box plot of progeny size of N2 compared to the sld-3(2E) MosSCI strain. **C-E**. As A, but with RNAi of *cye-1* (C), *cyb-1* (D) or *cyb-3* (E). *** P-value <0.005, NS = non-significant. P values were calculated using a Mann whitney-Wilcoxon test.
(EPS)

S3 Fig SLD-3 and SLD-2 are asymmetrically localised in the early embryo. **A**. Box plot of SLD-3 mCherry signal ratio between the P1 and the AB cell. **B.** Images from A. Images show brightfield, Hoescht and mCherry signal of the wild type (N2) or the respective MosSCI lines. mCherry signal corresponds to SLD-3 expression in the two-cell embryo. Scale bar is 10μm. **C**. Quantitation of SLD-2-GFP fluorescence from live imaging. Graph shows fluorescent intensity signal of the AB and P1 cell. The graph represents one-minute time-lapse movies of two cell embryos. The time point 0 is the nuclear envelope breakdown (NEB) in the AB cell. Error bars are 95% CI; n = 8 **D.** Images of SLD-2 GFP taken from a late one-cell embryo, after the nuclei reform but before cytokinesis and also 2 minutes later. Scale bar is 10μM.
(EPS)

S4 Fig Generation of PKC-3 interaction mutant in SLD-2. **A.** Alignment of the C-termini of SLD-2 proteins from the indicated nematode species. The region of interaction between *C*. *elegans* SLD-2 and PKC-3 is indicated in red. **B**. Summary of the yeast two-hybrid interactions between SLD-2 mutants and PKC-3 1–219. **C**. Yeast two-hybrid growth assays of some of the mutants described in B. **D**. Images of PKC-3 GFP and SLD-2 mCherry. This is the same image as Fig 6D, but with increased brightness of the mcherry signal to visualise SLD-2 in the cytoplasm. Scale bar is 10 μm.
(EPS)

S5 Fig Cell cycle lengths of the *sld-2(EDDD)* mutant. **A.** Box plot of the AB and P1 cell cycle length from the indicated strains. Cell cycle length was calculated as the time from the P0 cell nuclear envelope breakdown (NEB) to the AB or P1 NEB from 8s time-lapse movies. p-values were calculated using the Wilcoxon test. B. Delay between AB nuclear envelope breakdown (NEB) and P1 cell NEB for the indicated strains, with and without RNAi of rif-1. There are 2 isoforms of rif-1 in *C*. *elegans* (F11E6.7 and F19H6.3), which we have named here a and b. The RNAi was an equal mix against both isoforms.
(EPS)
